# The power of citizen science to advance fungal conservation

**DOI:** 10.1111/conl.13013

**Published:** 2024-03-22

**Authors:** Danny Haelewaters, C. Alisha Quandt, Lachlan Bartrop, Jonathan Cazabonne, Martha E. Crockatt, Susana P. Cunha, Ruben De Lange, Laura Dominici, Brian Douglas, Elisandro Ricardo Drechsler-Santos, Jacob Heilmann-Clausen, Peter J. Irga, Sigrid Jakob, Lotus Lofgren, Thomas E. Martin, Mary Nyawira Muchane, Jeffery K. Stallman, Annemieke Verbeken, Allison K. Walker, Susana C. Gonçalves

**Affiliations:** 1 Department of Ecology and Evolutionary Biology, University of Colorado Boulder, Boulder, Colorado, USA; 2 Faculty of Science, University of South Bohemia, České Budějovice, Czech Republic; 3 Biology Centre of the Czech Academy of Sciences, Institute of Entomology, České Budějovice, Czech Republic; 4 Department of Microbiology and Infectious Diseases, Université de Sherbrooke, Sherbrooke, Québec, Canada; 5 Forest Research Institute, Université du Québec en Abitibi-Témiscamingue, Amos, Canada; 6 Centre for Forest Research, Université du Québec à Montréal, Montréal, Canada; 7 Leverhulme Centre for Nature Recovery, School of Geography and the Environment, University of Oxford, Oxford, UK; 8 Centre for Functional Ecology, Associate Laboratory TERRA, Department of Life Sciences, University of Coimbra, Coimbra, Portugal; 9 Royal Botanic Gardens, Kew, Richmond, UK; 10 Research Group Mycology, Department of Biology, Ghent University, Ghent, Belgium; 11 Department of Environment, Land and Infrastructure Engineering, Politecnico di Torino, Turin, Italy; 12 Research Group MIND.Funga, Departamento de Botânica, Universidade Federal de Santa Catarina, Florianópolis, Santa Catarina, Brazil; 13 Center for Macroecology, Evolution and Climate, Globe Institute, University of Copenhagen, Copenhagen, Denmark; 14 School of Civil and Environmental Engineering, University of Technology Sydney, Sydney, Australia; 15 Fungal Diversity Survey, Sebastopol, California, USA; 16 Department of Biology, Duke University, Durham, North Carolina, USA; 17 School of Natural Sciences, College of Environmental Sciences and Engineering, Bangor University, Bangor, Wales, UK; 18 Department of Botany, National Museums of Kenya, Nairobi, Kenya; 19 Department of Botany and Plant Pathology, Purdue University, West Lafayette, Indiana, USA; 20 Department of Biology, Acadia University, Wolfville, Nova Scotia, Canada

**Keywords:** amateurs, extinction risk, fungal distribution, iNaturalist, mycology, online databases, Red List

## Abstract

Fungal conservation is gaining momentum globally, but many challenges remain. To advance further, more data are needed on fungal diversity across space and time. Fundamental information regarding population sizes, trends, and geographic ranges is also critical to accurately assess the extinction risk of individual species. However, obtaining these data is particularly difficult for fungi due to their immense diversity, complex and problematic taxonomy, and cryptic nature. This paper explores how citizen science (CS) projects can be lever-aged to advance fungal conservation efforts. We present several examples of past and ongoing CS-based projects to record and monitor fungal diversity. These include projects that are part of broad collecting schemes, those that provide participants with targeted sampling methods, and those whereby participants collect environmental samples from which fungi can be obtained. We also examine challenges and solutions for how such projects can capture fungal diversity, estimate species absences, broaden participation, improve data curation, and translate resulting data into actionable conservation measures. Finally, we close the paper with a call for professional mycologists to engage with amateurs and local communities, presenting a framework to determine whether a given project would likely benefit from participation by citizen scientists.

## 1 ∣ INTRODUCTION

Citizen science (CS) consists of engaging the public in research practice and scientific knowledge generation, empowering nonscientists to generate reliable data that can be used by academics, policymakers, and other stakeholders (Miller-Rushing et al., 2012). Although such nonexperts have contributed to scientific discourse for centuries (Miller-Rushing et al., 2012; Silvertown, 2009), the term “citizen science” first appeared only in 1989. Kerson (1989) provided a preliminary description of the practice of generating data through the engagement of nonexpert volunteers and nonprofessional scientists (e.g., amateur naturalists) in order to address politically or socially relevant issues. This concept has since evolved so rapidly that today it has become difficult to provide a single definition (Haklay et al., 2021). Here, we use the term to refer to public engagement in scientific research, in which community members actively contribute to science either intellectually or with tools and resources.

The growth of CS has been fostered by emerging information technologies and social media platforms that have significantly simplified data acquisition and data transfer among involved parties (Newman et al., 2012). These modern technologies present opportunities to further democratize science by providing nonexperts access to scientific information and promoting global information-sharing among amateurs and professionals. Indeed, among the major advantages of CS is that individuals with varying levels of expertise, ability, and time can participate (Irga et al., 2020).

CS projects are becoming increasingly utilized in various scientific fields, such as medicine, ecology, meteorology, and public health (Lewandowski et al., 2017), and are an increasingly powerful means to collect diverse forms of biological data (Bonney, 2021; Silvertown, 2009). These data have been used to explore questions related to species distributions (e.g., van Strien et al., 2013), demographic trends (Horns et al., 2018), the spread of invasive species (Brown et al., 2001), and the value of ecosystem services (Harrison et al., 2020). In mycology, CS initiatives are helping to address specific challenges such as high species diversity, unresolved taxonomy, and conservation (Heilmann-Clausen et al., 2019; Koukol et al., 2020; Polemis et al., 2023). In this review, we summarize the achievements of CS in the study of fungal diversity to date and explore possible future applications. We also highlight key challenges to CS and discuss ways to overcome them.

## 2 ∣ HARNESSING CITIZEN SCIENCE TO ADVANCE FUNGAL CONSERVATION

**FIGURE 1:**
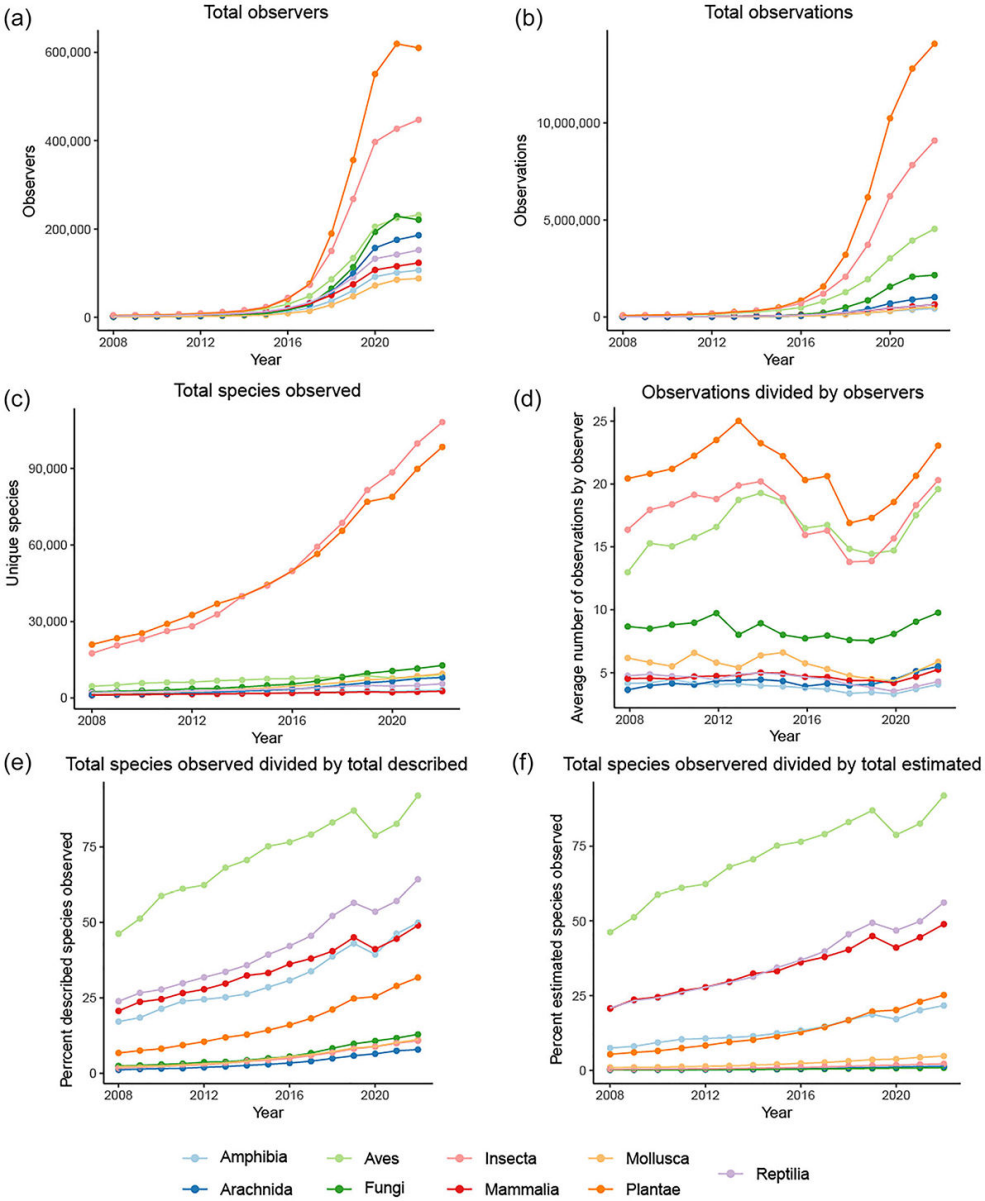
Annual data on observations, observers, and species in nine organismal groups on iNaturalist.org from 2008 to 2022: (a) total observers; (b) total observations; (c) total unique species observed; (d) average number of observations by observer; (e) percentage of total described species observed; (f) percentage of total estimated species observed. Total described and estimated species in (e) and (f) derived from https://www.dcceew.gov.au/science-research/abrs/publications/other/numbers-living-species/executive-summary.

Fungi are ubiquitous and fulfill diverse ecological roles. They are involved in countless interactions with other organisms, including plants, animals, bacteria, protists, and other fungi. Although the estimated number of fungal species is approximately 2.5 million (Niskanen et al., 2023), only around 154,000 are currently described (Bánki et al., 2023). This biological knowledge shortfall has impeded global fungal conservation efforts across different scales (Hortal et al., 2015; Haelewaters et al., 2024). Current biological conservation frameworks depend on observation and data collection. Thanks to molecular data, our capacity to monitor fungal biodiversity has expanded to include poorly studied taxa that were historically over-looked with traditional field-based observations (Cazabonne et al., 2022). Despite this progress, our understanding of these groups remains limited by a lack of sampling across time and geography (Halme et al., 2012) (Figure 1).

Notwithstanding these gaps in knowledge, fungal conservation has gained momentum. The FF&F initiative, adding the term funga to be used alongside fauna and flora (Kuhar et al., 2018), aims to write fungi into conservation and agricultural policy frameworks and unlock funding for mycological research, surveys, and educational programs. Chile became the first country to specify that fungi should be included in formal conservation and management policies (República de Chile, 2010). Another milestone was the publication of open letters calling on policymakers to include all fungi in global biodiversity targets (Gonçalves et al., 2021) and acknowledge their roles in ecosystems (Palahí et al., 2022). Finally, an unequivocal mark of progress is the sharp increase in the number of assessed fungal species on the IUCN Red List of Threatened Species (hereafter the Red List) (Mueller et al., 2022), which grew from 2 in 2003 to 625 as of 27 June 2023 (International Union for Conservation of Nature [IUCN], 2023).

**FIGURE 2:**
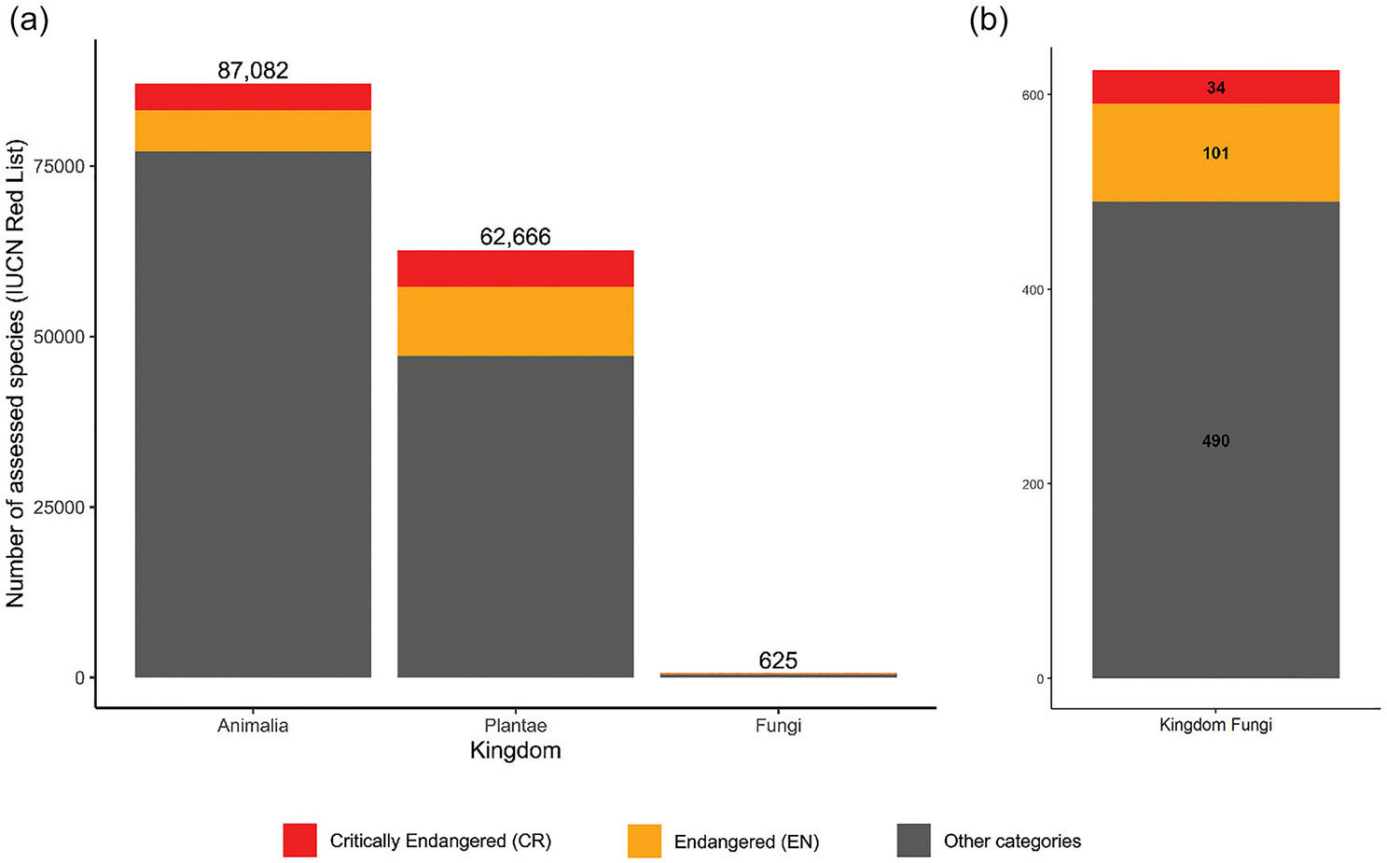
Number of species assessed on the IUCN Red List of Threatened Species (IUCN, 2023): (a) assessments completed for three eukaryotic kingdoms; (b) assessments completed for the kingdom Fungi. Data taken from https://www.iucnredlist.org/resources/summary-statistics.

Despite these advances, fungal conservation continues to face challenges. First, relative to many other taxa, fungi are often neglected in biological research due to their cryptic nature (Rambold et al., 2013). As such, the number of Red List assessments of fungi pales in comparison to other multicellular organisms (Figure 2). As of 27 June 2023, 87,082 animal species and 62,666 plant species have been assessed by the IUCN (2023), compared to just 625 fungi. Second, within fungi, there is a research bias towards groups that produce macroscopic fruiting bodies (Gonçalves et al., 2021). Third, given the multitude of their interactions, fungal conservation efforts inherently necessitate the conservation of their associated organisms, adding further complexity to these efforts (Tylianakis et al., 2010). Finally, there remains a lack of resources and personnel for expert-led fieldwork surveys—a trend that may intensify given the decline in prioritization of field-based studies among conservation scientists (Ríos-Saldaña et al., 2018).

One way to overcome these challenges and address fundamental issues in fungal conservation is to leverage CS-sourced data (Heilmann-Clausen et al., 2019). Projects involving local communities and amateur mycologists are becoming more popular as the scientific value of data collected by nonprofessionals is increasingly recognized (Silvertown, 2009). Given the limited number of professional mycologists and funding resources, opportunities exist for members of the public to provide significant contributions in accumulating large amounts of data in a short period (Gryzenhout, 2015). Amateur observers have the potential to generate numerous fungal records, including those of undescribed and endangered species that can be used to inform fungal conservation efforts (Irga et al., 2020).

Even small-scale CS efforts in mycology can provide valuable insights into fungal species distributions and ecological interactions by gathering images and mapping species occurrences (e.g., Heilmann-Clausen et al., 2016; Shumskaya et al., 2023). Amateur mycologists are often among the only sources of data collection in under-documented regions. Furthermore, interested amateurs could assist in searching for rare species under professional scientific guidance and discovering fungal species new to science (e.g., Crous et al., 2017, 2021).

## 3 ∣ EXAMPLES OF ONLINE DATABASES AND FUNGAL CITIZEN SCIENCE PROJECTS

In this section, we present the primary online databases used for data collation and several examples of fungal CS initiatives to showcase how they can be planned, structured, and used. Although not a comprehensive list, these examples illustrate the potential of CS-generated datasets to examine fungal biodiversity trends and distributions across landscapes and time. We categorize projects by organizational structure: unstructured projects, targeted approaches, and derived projects.

### 3.1 ∣ Online databases

Digital occurrence data can be gathered on a large scale using online repositories. Such tools can be very powerful with respect to observing distribution patterns. Although some projects have developed their own specific repositories, iNaturalist and Mushroom Observer are the most widely used CS online databases in mycology.

iNaturalist (https://www.inaturalist.org/) is an online CS platform that depends on community identification of submitted records. Anyone globally can submit observations, usually images of an organism taken in the field. The platform can suggest identifications based on a computer vision model. However, only those observations with sufficient metadata and identifications confirmed by other users are considered “research grade.” This not only encourages users to interact with each other but also functions as a quality filter (Hiller & Haelewaters, 2019). The value of such records to conservation relies on their volume and accessibility; all that is needed to contribute is a suitable device and internet access. Projects can be created on iNaturalist to aggregate observations based on specific criteria such as location and taxon. For example, iNaturalist data have been used to reevaluate the common discomycete genus *Bisporella* (Mitchell et al., 2022) and to update the geographical distribution of the biotrophic microfungus *Hesperomyces harmoniae* (de Groot et al., 2024). As of 28 June 2023, iNaturalist has recorded 8,741,713 observations representing 20,096 species of fungi submitted by 651,795 contributors.

Before the existence of iNaturalist, Mushroom Observer (https://mushroomobserver.org/) served as the primary international platform for recording CS fungal observations online. It is still used by citizen scientists for recording sightings, publishing images, and identifying fungi. Thus far, Mushroom Observer collates 480,069 observations, representing 18,923 taxa recorded by 11,641 contributors. Professional mycologists using these online CS databases can access huge amounts of data; for example, Bazzicalupo et al. (2022) calculated that iNaturalist and Mushroom Observer combined have resulted in over 500,000 CS observations of fungi in Canada alone.

The Global Biodiversity Information Facility (GBIF, https://www.gbif.org) is an open-access platform that aggregates occurrence records from multiple sources. “Research grade” observations from iNaturalist are automatically uploaded to GBIF. Records from specific CS projects (see below) and fungaria can also be submitted to GBIF. These CS observations are then made more widely available for scientific use, increasing the completeness of species distribution maps needed for conservation work.

### 3.2 ∣ Unstructured projects

Unstructured CS projects form part of broad recording schemes. These projects aggregate observations through online databases, by digitizing foray collections, or from dried specimens sent to professional mycologists for validation. Below, we present several CS projects, from the local up to the continental scale.

To map fungal diversity in and around the city of Coimbra, Portugal, the local CS project Cogumelos na Cidade (Mushrooms in the City) uses iNaturalist’s “Projects” tool as a platform (https://www.inaturalist.org/projects/cogumelos-na-cidade). As of 28 June 2023, 148 contributors have contributed 2232 observations, representing 365 species, of which 223 are “research grade.” Although it only started in September 2020, this project has already resulted in the identification of new records for Portugal and helped document the presence and spread of exotic species. Vouchers for several of these observations await identification and will likely contribute to additional new and rare species for Portugal.

The New Jersey Mycological Association collections from regional forays over 12 years were collated into a dataset containing 400,260 occurrences of 1483 species (Shumskaya et al., 2023). Approximately 3000 specimens are curated in a private herbarium at Rutgers University. The database is georeferenced and accessible through GBIF.

The MIND.Funga App (https://mindfunga.ufsc.br/app/?lang=en) is the latest development of a regional CS project that was started in 2020 in southern Brazil to record occurrences of fungal species with images and associated metadata. It represents a tool for macrofungal recognition based on submitted images, identified using a deep learning neural network model. Species names are suggested with a confidence rating. Thus far, the MIND.Funga App has received 17,467 images representing more than 500 species (Drechsler-Santos et al., 2023).

The Danish Fungal Atlas (https://svampe.databasen.org/en/) is a national CS project aiming to build a checklist of macrofungi in Denmark. Any individual can submit data on location and associated organisms of rare and common fungi. Records are cross-referenced with GBIF. In total, 5014 observers have contributed 1,109,146 expert-validated records, representing 9081 species. Of those, 15 were new to science, and 197 were new records for Denmark (Heilmann-Clausen et al., 2019). Several research papers have used these data to explore subjects such as host selection in wood-inhabiting fungi (Heilmann-Clausen et al., 2016), biases in recording schemes in CS (Geldmann et al., 2016), and biodiversity patterns (Andrew et al., 2018). This project has also informed systematic conservation planning in Denmark (Petersen et al., 2016) and supported the development of AI-based identification tools (Picek et al., 2022).

The “Mushrooms of Kenya” project was initiated in 2019 to create awareness about fungi among local communities and conservationists, develop a countrywide field guide, and improve understanding of fungal distribution patterns. An iNaturalist project (https://www.inaturalist.org/projects/mushrooms-of-kenya) was created to collect observations of macrofungi in Kenya. As of 28 June 2023, 294 contributors have made 4240 observations, representing 424 species. In addition, 3488 specimens are being maintained at the East African Herbarium, National Museums of Kenya. The project has an educational program that equips CS participants with training in macrofungal sampling, preservation, and identification.

Fungimap (https://fungimap.org.au/) is an Australian CS organization hosting an iNaturalist project (https://inaturalist.ala.org.au/projects/fungimap-australia) with 90,386 observations representing 1768 species recorded by 914 contributors, as of 28 June 2023. The National Australian Fungimap Database was established to track rare and threatened species and currently has more than 100,000 records provided by 1000 contributors. Fungimap also collaborates with BioSMART to host the Great Aussie Fungi Quest (https://www.biosmart.life/australian-fungi-quest-2023). This is an annual event similar to the Great North American Fungi Quest but only runs on two apps: QuestaGame.com and iNaturalist.org. As of 28 June 2023, 13,309 observations representing 1131 species were recorded by 1973 contributors.

Finally, as an example of a continental CS project, the Great North American Fungi Quest is a month-long fungal survey (https://www.biosmart.life/north-america-fungi-quest-2022). Participants submit observations through one of six CS apps: iNaturalist.org, MushroomObserver.org, Observation.org, Questagame.com, NatureSpots.net, and CitSci.org. Aggregating data from multiple apps increased accessibility, which resulted in 157,701 observations representing 7612 species recorded by 34,532 people in 2022.

### 3.3 ∣ Structured approaches

Structured CS projects provide participants with targeted sampling approaches, protocols, or species lists. Advantages of such projects include allowing researchers to streamline data collection toward addressing specific questions, challenges, and conservation goals. Examples of structured projects include the FunDiS Rare Challenges, Lost and Found Fungi, and the Distribution Research Forest Mushrooms of the Dutch Mycological Society.

The Fungal Diversity Survey (FunDiS) hosts a biodiversity database through iNaturalist (https://www.inaturalist.org/projects/fundis-biodiversity-database) and has launched several Rare Challenges (https://fundis.org/protect) to document rare and threatened fungi from a list of target taxa in specific geographic areas. FunDiS makes posters and pamphlets available to participants of Rare Challenges, with relevant information about target taxa, such as preferred habitat, seasonality, potential range, and similar-looking species. The West Coast Rare Fungi Challenge focuses on 20 species of macrofungi and, since its inception in October 2020, has amassed 681 observations representing 16 species submitted by 388 contributors. Thus far, different FunDiS initiatives have resulted in 183,932 observations in iNaturalist, representing 5691 species submitted by 1425 contributors as of 8 November 2023.

The Lost and Found Fungi (LAFF) project (https://fungi.myspecies.info/content/lost-and-found-fungi-project) was hosted by the Royal Botanic Gardens, Kew from 2014 to 2019 to produce a dataset of occurrences for rare target species not observed for ≥50 years. Records taken from the literature and fungaria were supplemented by submissions from citizen scientists. This project resulted in approximately 1400 new records representing 77 species and facilitated Red List assessments of 20 target species.

The Dutch Ecological Monitoring Network (https://www.netwerkecologischemonitoring.nl/) is government-run and has programs across organismal fields, including the Mushroom Monitoring Program (Meetnet Paddenstoelen) of the Dutch Mycological Society. It consists of three different projects, for which volunteers collect data. Since 1998, the Verspreidingsonderzoek Bospaddenstoelen (Forest Mushrooms Distribution Research) aims to document 138 typical species annually in forests on sandy soils and dunes (https://www.verspreidingsatlas.nl/projecten/nmv/bospaddenstoelen/). Each 1 × 1 km^2^ plot is monitored three times annually, with participants recording all mushrooms observed and corresponding metadata along a fixed 0.5–1.0 km-long transect. Expert users verify all submitted observations.

### 3.4 ∣ Derived projects

Another way to involve CS participants in gathering fungal biodiversity data is to have them submit environmental (i.e., non-fruiting body) samples from which fungi can be obtained or sequenced. These so-called derived projects require extra steps by professional mycologists to “derive” fungal biomass or sequences from the samples. One example is the Dutch garden soil school project “Wereldfaam, een schimmel met je eigen naam” (World fame, a fungus with your name). It used 404 soil samples submitted by children to obtain 4750 fungal isolates and has thus far resulted in the description of 2 new genera and 24 new species of *Mucoromycota, Dothideomycetes, Eurotiomycetes, Leotiomycetes*, and *Sordariomycetes* (Crous et al., 2017, 2021; Hou et al., 2020). A second example is FunLeaf (https://sisu.ut.ee/funleaf/about), which seeks to describe global leaf endophyte communities using leaves submitted by CS participants. Detailed protocols are provided for submission, and all data are made available to participants through the PlutoF Biodiversity Platform (https://plutof.ut.ee/).

## 4 ∣ CHALLENGES AND SOLUTIONS

CS is uniquely positioned to capture biodiversity data across spatial, temporal, and taxonomic axes on a scale often unachievable for professionals. Indeed, the above examples showcase evidence that CS is producing useful output for mycologists. However, to truly harness the power of CS to advance fungal conservation, a number of obstacles spanning data curation to community engagement must be addressed. Here, we present five challenges and provide recommendations to improve the utility and impact of CS for fungal conservation.

### 4.1 ∣ Capturing fungal diversity

One of the greatest challenges in CS is how to overcome taxonomic biases, particularly concerning fungi that are cryptic, ephemeral, and speciose. Public records are heavily biased by organismal preference (Figure 1). Most CS projects focus on large, easily identifiable sporocarps and, as such, document only a fraction of extant fungal diversity. CS projects targeting microfungi, by contrast, need to provide advanced training and equipment because these fungi are difficult for non-specialists to find and identify. Similarly, fungi requiring microscopy and chemical testing for proper identification are a challenge for inclusion in CS projects. As an example, McMullin and Allen (2022) found that the proportion of accurate identification of lichen species on iNaturalist.org was 59% when only macromorphology was needed but dropped to as low as 5% when further analyses were required.

Targeted projects can increase awareness and appreciation of understudied fungal groups. Successful targeted projects have resulted in many scientific contributions, including the identification of new species (Koukol et al., 2020) and insights into how environmental contamination may affect the development of endangered fungal species (Irga et al., 2018). CS participant-led initiatives, such as the Welsh Microfungi Group, are building interest and momentum around underappreciated fungal groups, including microfungi and pathogens such as downy mildews and parasitic hyphomycetes (Chater et al., 2021).

To minimize low-confidence observations, experts could pre-identify which species require information beyond a macroscopic image. Taxa requiring microscopy, DNA, or host information for an accurate identification could be indicated in CS repositories. Observers of these species would then need to indicate whether specialized data beyond macroscopic images were supplied. Observations lacking these specialized data would not be considered “research grade.” Professional mycologists can also play a crucial role as identifiers on CS platforms by helping to identify and comment on observations made by other users or downgrading observations that do not meet the needed criteria.

To identify difficult species, CS participants may benefit from automated image recognition models used in platforms such as iNaturalist, although these still have limitations. The accuracy of machine learning (ML) models is predicated on training data and requires sufficient and accurate examples (Picek et al., 2022). ML-based identification therefore risks perpetuating inaccurate identifications if based on biased input samples and likely makes automated image identification less useful for under-sampled taxa (Koch et al., 2022). Because ML models depend on the presence of distinguishing features, species that lack identifiable characters can confound accurate identification for CS participants and ML models alike. Nevertheless, ML has great potential, and the increased availability of benchmarked training data along with the proliferation of new, increasingly complex models are expected to improve the utility of ML in species identification.

Finally, environmental DNA (eDNA) approaches can be more sensitive in capturing total fungal diversity than those based on sampling individual fruiting bodies (Nilsson et al., 2019; Shirouzu et al., 2020), although using eDNA has its own caveats (Carini et al., 2016). Projects utilizing CS participants to gather environmental samples, such as soil, litter, wood, and other plant material, for eDNA extraction and analysis are well poised to help capture a more complete view of fungal biodiversity. The FunLeaf project, mentioned above, is one example already employing CS in these efforts.

### 4.2 ∣ Estimating species absences

An important element in assessing biodiversity for conservation is our ability to identify where species are truly absent (Martin et al., 2023). Although presence-only data is widely considered to be inferior to presence–absence data for determining species distributions (Johnston et al., 2021), proving true absence is more difficult than proving presence, particularly in cryptic taxonomic groups (e.g., Haelewaters et al., 2024; Kéry, 2002). As such, current fungal repositories and datasets consist almost exclusively of presence-only observations.

CS fungal repositories could aid in tracking absence data by incorporating the ability to state whether the submitted observation is part of a “complete list” of all fungi found for the location visited, as is currently possible in eBird and eButterfly (Johnston et al., 2021; Prudic et al., 2017). Ideally, such a feature would allow for editable definitions of what a “complete list” entails based on individual sampling schemes (e.g., allowing users to state that they recorded all poroid fungi on woody substrates over 1 cm in diameter). For complete lists, a function for reporting effort (such as time spent and number and experience level of participants) would further enable confidence scoring both presence and absence data (Johnston et al., 2021; Picek et al., 2022).

Structured projects can help document absences by arming CS participants with informed search lists for target species—a concept that has also been applied to other taxa (Martin et al., 2023). The continued absence of taxa, even after targeted search campaigns, adds to our understanding of true absence and species rarity. This was the case for *Hypocreopsis amplectens*, now considered critically endangered. Researchers were able to nominate the species for protective status after it spent many years on the 100-species Fungimap target list and yielded only 13 observations (Buchanan & May, 2019). It should be noted that absence-only data can also be a valuable contribution to conservation. After Sweden’s ash populations were devastated by the fungus *Hymenoscyphus fraxineus*, the Rädda Asken (Save the Ash) CS program was created to locate healthy trees that lacked signs of *H. fraxineus* infection, successfully identifying hundreds of genotypes for resistance breeding (https://raddaasken.nu/en/home; Hulbert et al., 2023).

Recording and monitoring fungal distributions and rarity are complicated by the phenology of fruiting (which may occur seasonally) and ephemerality of fungi and their substrates (Straatsma et al., 2001). When the fruiting of a given species does not occur year-round or every year, absences of that species in CS projects may be difficult to interpret. The ephemeral nature of sporocarps also represents a challenge when recording diversity (Cazabonne et al., 2022; Kauserud et al., 2008), exacerbated when hosts or habitats are ephemeral themselves. Examples of this include species of *Hypomyces* that parasitize mushrooms in *Agaricales* (Rogerson & Samuels, 1994) and *Pyxidiophora* that parasitize fungi growing on herbivore dung and decomposing plant materials (Haelewaters et al., 2021). Finally, ease of access to collection sites is another factor adding geospatial bias to observation datasets. For example, on large scales, CS projects tend to source more data from nontropical countries and more accessible and densely populated parts of nontropical countries (Tiago et al., 2017), whereas on more local scales, sampling is often concentrated around easily accessible trails (Mandeville et al., 2022).

### 4.3 ∣ Broadening participation

Ensuring CS participation from people with a diversity of backgrounds is critical to both equitable community engagement and unlocking the full potential of CS to inform fungal conservation. Recruiting and retaining participants from diverse backgrounds can help fill geospatial gaps, provide experts with knowledge of endemic taxa, and increase the investment of various people and communities interested in fungal conservation. Unequal engagement across socioeconomic and sociopolitical axes currently leads to overrepresentation of sampling in North America, Europe, and Australia, while leaving taxa in vast stretches of the world under-reported and under-characterized (Lofgren & Stajich, 2021; Quandt & Haelewaters, 2021). Gaps in CS engagement currently mean lower participation among people of color, women, and low-income earners (Pateman et al., 2021). Developing strategies toward broader engagement should be a priority for the field.

Day et al. (2022) found that enthusiasm to engage in future conservation efforts is only expressed in participants who feel they made a significant contribution to a CS project. In this way, project assessment and reporting are critical to repeated engagement and improving the design of future programs. In summary, CS projects should (1) clearly and continuously communicate project goals, (2) strive for diverse representation among leaders and participants, (3) account for participant experience level, (4) provide various engagement opportunities for people experiencing different motivations and barriers, (5) share final project results with participants, (6) obtain feedback from CS participants, and (7) publish a formal report on participant assessments (Cheeke et al., 2018; Estes-Zumpf et al., 2022; Jordan et al., 2012; Kaplan Mintz et al., 2023; Lofgren & Stajich, 2021).

### 4.4 ∣ Improving data curation

Hundreds of thousands of contributors have submitted millions of fungal observations on CS platforms. These represent a variety of data types, including primary and secondary image data, location data, sequence data, and a variety of metadata formats ranging from qualitative text-based descriptions to linked projects and confidence scores. Multiple efforts are underway to maximize the utility of this influx of information and link diverse data types across platforms for informative use and reuse. Managing data quality at this scale is another point of concern, because participant experience, community feedback, and curation vary widely. Beyond platforms designed for CS participation, numerous web-based applications curate fungal observations that are presently largely untapped for research. For example, the Facebook Group “Mushroom Identification” has 359,709 members who have posted over 1 million photos as of 28 June 2023.

Data should be openly accessible and presented in a widely usable format. Mechanisms for the procurement of flat files are available on most major platforms (e.g., via the “Get Data” filters built into GBIF, which can further link downloaded observation lists with a DOI for research publications). Many such platforms include options to include multiple data types directly, along with options to cross-list data housed on different platforms. For example, iNaturalist.org also includes fields for barcode sequences and vouchering information that can be linked to other databases (e.g., NCBI GenBank, MyCoPortal) and resultant publications. Even when observations lack metadata, they can be useful for a variety of purposes, such as phenology and image mining through ML. Therefore, it is critical that all CS projects are preserved and publicly accessible for reuse, including unprocessed raw data packaged under a stable DOI in a long-term repository such as Data Dryad (https://datadryad.org/) or Zenodo (https://zenodo.org/), in accordance with the FAIR data principles (Lofgren & Stajich, 2021; Wilkinson et al., 2016). To increase the accessibility of data posted to alternative platforms such as social media groups, we encourage group moderators to promote cross-posting to CS repositories, which could also be facilitated via third-party apps.

### 4.5 ∣ Translating data into action

The conservation of fungi lags behind that of other organismal groups (Gonçalves et al., 2021; Lofgren & Stajich, 2021) (Figure 2). Building support and understanding around fungal conservation is a significant challenge that must be addressed with multiple stakeholders, including the public, policymakers, governments, and funding agencies. Public campaigns such as those developed by the Fungi Foundation (https://www.ffungi.org) have helped build appreciation for fungal conservation among the general public, but interest from policymakers and funding agencies remains meager. Research funding for taxonomists is low (Britz et al., 2020) and related to a dearth in academic positions in fungal taxonomy. CS projects can help fill some of these funding gaps by providing community-sourced data, but monetary assistance is still needed to support the scientists and institutions leading these initiatives.

CS project leaders and CS databases should prioritize the collection and accessibility of data necessary for Red List assessments, including range descriptions and abundance estimates that include the ability to infer true absences. To translate Red List status into legal protection, a species must be included in legislation. However, the manner and extent of protection can vary drastically among different countries. In the United States, for example, the Endangered Species Act (ESA) is widely regarded as the most significant piece of policy regulating species protection. As of 27 June 2023, of the 1870 species currently considered endangered under the ESA, only 3 are lichens, and none are free-living fungi (https://ecos.fws.gov/ecp/report/taxonomic-list-tess).

## 5 ∣ ENGAGING CITIZEN SCIENCE

Here, we present a call for professional mycologists to engage with amateur naturalists and local communities to contribute knowledge of fungal diversity, distributional ranges, phenology, and occurrence data to inform Red List assessments of species. Professional researchers may feel uneasy about relying heavily on CS participants for their research, due to concerns about data quality and metadata completeness, the added professional demands created by working with amateurs, and lack of funding. Although it is important to strive for best practices regarding observations and fungarium collections, these should not exclude much useful data and or dissuade beginners, students, and amateurs from getting involved or sharing observations and collections.

Some projects are more amenable to CS participation than others. To help professional mycologists decide whether their projects may benefit from CS, we have constructed a dichotomous key based on the decision framework by Pocock et al. (2014):

1. Some or all aspects of project can be completed entirely online............................suitable for CS

1′. No aspects of project can be completed online..................................go to 2

2. Project involves collection of something too small to be seen with naked eye.......................go to 3

2′. Project involves collection of something visible to naked eye...............................go to 4

3. Project leader has ability to train participants in proper sampling (e.g., with design of pamphlets or videos).....................................................go to 4

3′. Project leader does not have ability to train participants in proper sampling...............not suitable for CS

4. Sites are located where people frequently go...go to 5

4′. Sites are not located where people frequently go........................if properly incentivized, go to 5

5. Participants need authorized access to field sites to collect..........if proper permitting can be obtained, go to 6

5′. Participants do not need authorized access to field sites to collect............................go to 6

6. Collection or observation of samples requires complex protocols (including special equipment) not suitable for CS

6′. Collection or observation of samples does not require complex protocols..............................go to 7

7. Individuals require special expertise to accurately identify collections or observations..............................go to 8

7′. Individuals do not require special expertise to identify collections or observations...............suitable for CS

8. Expert volunteers are readily available to work on project.................................suitable for CS

8′. Expert volunteers are not readily available to work on project.............................go to 9

9. Project leader has ability to invest in training............................................suitable for CS

9′. Project leader does not have ability to invest in training......................not suitable for CS

## Data Availability

No new data were collected during the writing of this paper.
